# A Novel Model for Evaluating the Natural Antioxidant Carnosic Acid to Improve the Stability of Rapeseed Oil in the Thermal Degradation

**DOI:** 10.3390/antiox13030296

**Published:** 2024-02-28

**Authors:** Yingdan Zhu, Chengliang Chai, Yalin Xue, Yong Wang, Zhangqun Duan

**Affiliations:** 1 Institute of Cereal and Oil Science and Technology, Academy of National Food and Strategic Reserves Administration, Beijing 100037, China; 2 School of Chemical Engineering, The University of New South Wales, Sydney, NSW 2052, Australia

**Keywords:** rapeseed oil, carnosic acid, thermal degradation, model, chemical composition, viscosity

## Abstract

The quality and stability of oil during thermal processing reflect the reactions in vegetable oil. The deterioration of the oil is close to the viscosity, fatty acid composition (FA), total polar compounds (TPC), etc. Carnosic acid (CA) is the main antioxidant component of rosemary extract; it is a natural and clean-label antioxidant that is allowed to be added to prolong oil processing and storage. To achieve a clear correlation of this situation, a novel stability evaluation model was used to predict the thermal degradation of rapeseed oil (RSO) with CA. The RSO with CA (200 mg/kg, 400 mg/kg, and 700 mg/kg), the *tert*-Butylhydroquinone (TBHQ, 200 mg/kg), and the fresh RSO (without additives) during thermal processing (180 ± 5 °C) were studied. The temperature dependency of viscosity fits well with the *Lioumbas* model (*R*^2^ ≥ 0.999). The parameter *b* value in the *Lioumbas* model showed a decrease linearly with the processing time (*t_P_*, *R*^2^ ≥ 0.965). The multiple linear regression analysis showed that the accuracy of the model in predicting viscosity was less than ±2 mPa·s^−1^, and the deviation% was less than ±10% in all the samples. After 32 h of thermal degradation, the addition of 700 mg/kg CA showed the lowest degradation rate (13.84%) of polyunsaturated fatty acids (PUFAs), and the TPC content was 26.00 ± 0.50%. The TPC showed a positive relationship with viscosity (*r* = 0.99, *p* < 0.01), *t_P_* (*r* = 0.97, *p* < 0.01), and effective carbon numbers (ECN, *r* = 0.84, *p* < 0.05). In conclusion, this study can make a potential prediction for the stability of RSO.

## 1. Introduction

Rapeseed oil (RSO) is rich in high oleic acid unsaturated fatty acid, and it showed more oxidative stability in thermal degradation [1,2]. Oil degradation is the most important challenge in large-scale industrial processing. Using vegetable oil for deep frying is not only widely popular in China; it is also enjoyed in North and South America, Europe, and Africa [3]. Commonly, the heating temperatures are usually above 180 °C with the presence of oxygen. Numerous reactions occurred during the deep frying of vegetable oil, such as hydrolysis, oxidation, polymerization, and so on [4,5]. Some of these reaction products are volatile components and provide benefits by improving the flavor, taste, and color of food products. Over extended periods of processing, the stability of the vegetable oils can deteriorate, leading to an increase in foaming, higher viscosity, and high polymerization. Some reactions produce toxic compounds, such as total polar compounds (TPC), aldehydes, etc., which pose a threat to oil safety and food safety [6,7,8]. Long-term consumption can cause physical discomfort and even be linked to cancer [9].

The degradation of oil is the main factor threatening the quality and stability of oil. Parameters like the viscosity of oils determine their flow behavior, nutrient composition, and processability during frying [10]. The effective carbon number (ECN), as a function parameter, is based on the total number of carbons and the double bonds in triacylglycerol (TAG) molecules [11]. At present, TPC is used to determine the waste point of vegetable oil. This is the minimum allowable use of vegetable oils in the food industry. And the TPC value is reliable due to its accuracy and reproducibility [12]. In China, the TPC value is qualified as under 27%, and in many countries in the world, it is around 24–27% [13].

The relationship between thermal degradation and the quality of the oil has been the focus of different studies. Fasina and Colley (2008) focused on the vegetable oil type and viscosity with temperature, which showed the best fit with the modified *WLF* model [14]. There is a high linear correlation (*R*^2^ ≥ 0.942) between oil viscosity with C18:1 and C18:2 fatty acids (their double bonds) in 170 °C frying potato strips, according to the *Arrhenius* model [15]. For a better comparative analysis, four models have been studied (*Arrhenius*, *WLF*, *Arrhenius–Andrade*, and *Lioumbas*) in predicting the experimental profiles in palm oil and olive oil [16]. The above models can be used to evaluate the frying oils in a relatively short time and also could identify the waste point of vegetable oils. It also can simulate the process of oil degradation and prevent the production of oil hazards [17].

The application of antioxidants in oils can better protect the shelf life and protect against oil degradation [18,19]. The stability of vegetable oil has caused great concern among consumers. Rosemary *(Rosmarinus officinalis* L.) extract (RE), as a natural antioxidant, has been widely adopted as an effective way to delay oil oxidation [20]. In addition, 90% of the antioxidants in RE are relevant to the content of carnosic acid (CA) and carnosol (CN) [21]. It showed strong protection against peroxidation of palm oil under frying conditions [22]. Compared with the addition of BHT and BHA to soybean oils, RE had a better effect in protecting polyunsaturated fatty acids (PUFAs) content [23]. In terms of oil degradation in thermal processing, most current studies focus on antioxidant activity and oil-quality profile parameters, such as the fatty acid (FA) composition, acid value (AV), peroxide value (PV), *p*-anisidine value (*p*-AV), and inhibition of TPC, heterocyclic amines, polycyclic aromatic hydrocarbons, and trans-fatty acids in the vegetable oil [24,25]. However, few studies have investigated the correlation between CA addition and oil parameters and evaluated the model during thermal degradation. The viscosity parameters, together with TPC values, were used to predict and simulate experimental data during thermal degradation. The novel correlation between chemical composition changes, TPC value, ECN, and viscosity was evaluated to establish models for predicting the stability of RSO.

## 2. Materials and Methods

### 2.1. Materials

RSO was manufactured by COFCO Corporation (Beijing, China) without additives, and was stored at 4 °C before use. The carnosic acid (CA, 85%, PubChem CID: 65126) was purchased from Run Zekang Biotechnology Co., Ltd.(Beijing, China), and stored at 4 °C before use. Different concentrations of CA were added to RSO (200 mg/kg, CA-2, 400 mg/kg, CA-4, and 700 mg/kg, CA-7) for testing. The fresh RSO without additives was named the control sample (C), and the RSO with *tert*-Butylhydroquinone (TBHQ, PubChem CID: 16043, 200 mg/kg, TB-2) was used for comparison. The maximum addition of CA and TBHQ in vegetable oil followed the limitation set out by the Chinese Official Method (GB 2760-2014), i.e., 700 mg/kg and 200 mg/kg, respectively. Dissolution was accelerated by ultrasound (KQ-5200DE, Kunshan Shumei, Kunshan, China) for 20 min and with temperature control (≤50 °C). Fatty acid methyl ester mixture (18919-1AMP) was purchased from Sigma-Aldrich (St. Louis, MO, USA). The potassium hydroxide (PubChem CID: 14797) and sodium hydrogen sulfate (PubChem CID: 3423265) analytical grade (AR) were purchased from Sinopharm Chemical Reagent Co., Ltd.(Beijing, China). The Isooctane (PubChem CID: 10907) chromatographic grade (GR) was purchased from Fisher Scientific (Fair Lawn, NJ, USA).

### 2.2. Thermal Degradation Procedure

First, 100 g samples were added to the beaker. We put the samples into a high-temperature oven (DGG-9140AD, Shanghai Senxin, Shanghai, China) at 180 °C for nearly 0, 8, 16, 24, and 32 h before testing (take samples every 8 h) [26]. Each sample at each thermal condition was stored at −18 °C and triplicated for further analysis. For example, 200 mg/kg CA in RSO (CA-2) heating for 8 h was simplified as CA-2-8, and 400 mg/kg CA in RSO (CA-4) heating for 16 h was simplified as CA-4-16.

### 2.3. Viscosity Properties of Rapeseed Oil

Dynamic rheological properties were tested using (AR2000ex, TA Instruments, Waltham, MA, USA) a Peltier system to control the temperature. The rheometer was equipped with a 40 mm steel parallel plate. The viscosities of RSO were tested as a function of shear rate (10–1000 s^−1^) at 60 °C and 100 °C, respectively [27]. The temperature dependency of viscosity was tested at a shear rate of 50 s^−1^, with a temperature range from 60 °C to 110 °C. The heating rate of temperature was set at 5 °C/min. The correlation of heating temperature on viscosity (*μ*) can be measured by the *Lioumbas* model [16]:

ln*μ* = *a* + *b*(ln*T_m_*)^2^ (1)

where the parameters *a* and *b* are the constant data of the *Lioumbas* model, which is used as linear regression analysis; *μ* (mPa·s^−1^) is the viscosity of RSO; and *T_m_* (°C) is the measured temperature.

The typical *Arrhenius* equation was analyzed as a comparison model:

ln*μ* = ln*μ*_0_ + *E_a_*/R(*T_m_* + 273.15) (2)

where *μ*_0_ is the constant data; *E_a_* (J/mol) is the activation energy; and *R* is the universal gas constant, which is a constant parameter 8.314 J/mol·K.

### 2.4. Fatty Acid Composition of Rapeseed Oil

The fatty acid (FA) composition of RSO with CA during thermal degradation was determined following the Chinese Official Method (GB 5009.168-2016). The FAMEs were determined by a gas chromatograph (Agilent 7890B, Agilent Technologies, Santa Clara, CA, USA) equipped with flame ionization (SP-2560, 100 mm × 0.25 mm × 0.2 μm), and an auto-sampler injector was used. Nearly 60.00 mg of RSO was weighted, and it was measured according to a previous study [28].

It has been reported that the viscosities of the oils are related to the FA composition based on the TAG molecules [29]. The ECN can be used as a structural parameter, which consists of FA composition [30]. The equation for measuring the ECN is as follows:

ECN = ∑P_i_ (C_i_ −db_i_) (3)

where P is each FA composition (%); and C and db are the number of carbon atoms and the double bonds, respectively.

### 2.5. Total Polar Compounds (TPC) of Rapeseed Oil

The TPC content of RSO with CA in thermal degradation was tested using a Testo 270 rapid detecting instrument (270, Testo Inc., Lenzkirch, Germany) [31]. The instrument was calibrated using a standard oil sample of 3.5% at 55 °C. The deviation among all tests was less than ±0.5%.

### 2.6. Statistical Analysis

In this study, all the tests were performed in triplicates (mean ± standard derivatives). The temperature dependency of viscosity was analyzed by Origin 2021 (Origin Lab Corporation, Northampton, MA, USA) in both *Lioumbas* and *Arrhenius* mathematical models. Multiple linear regression analysis was calculated with the model coefficient from thermal degradation and chemical compositions of RSO with CA. The statistically significant difference (*p* < 0.05) was analyzed by IBM SPSS Statistics (Version 20.0, Chicago, IL, USA) and tested by Duncan’s test and *Pearson* correlation. All of the above figures in this work were illustrated using Origin 2021.

## 3. Results and Discussion

### 3.1. The Newtonian Behavior of Rapeseed Oil

**Figure 1:**
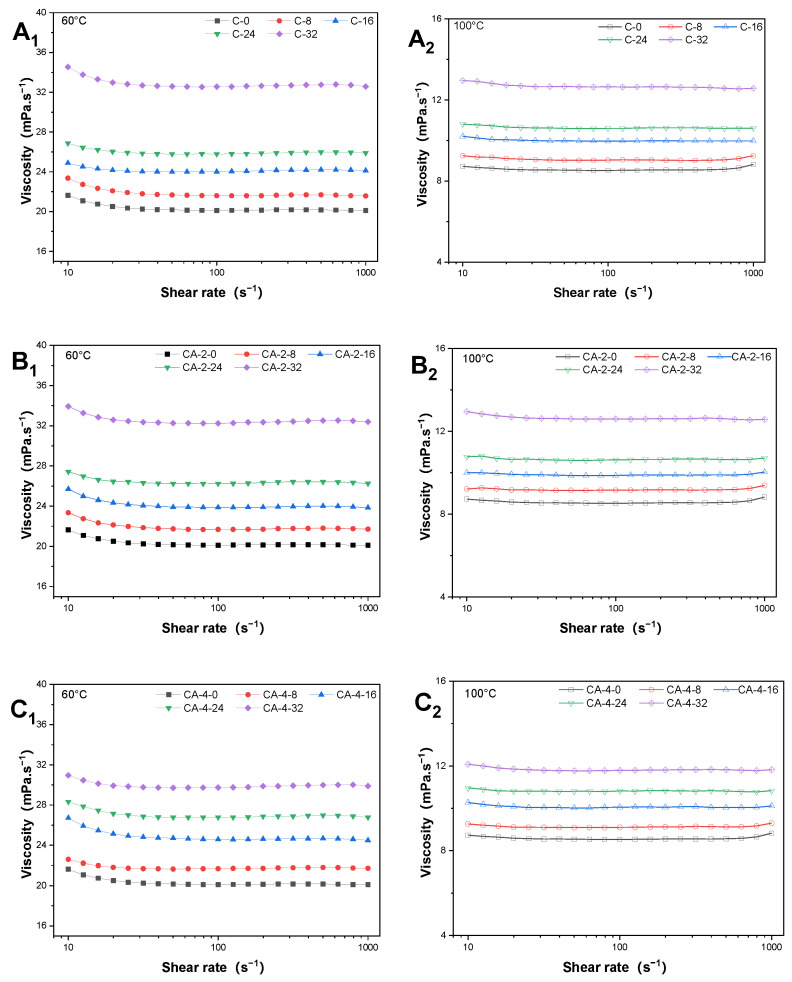

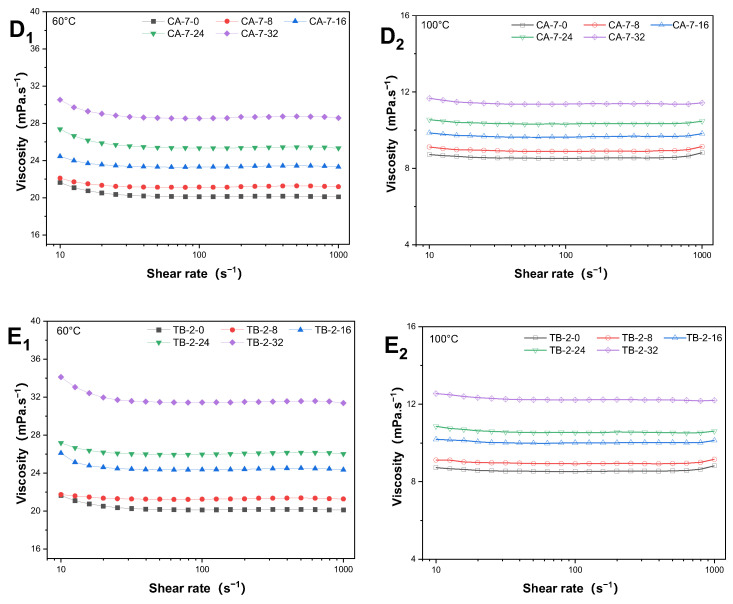
The apparent viscosity of rapeseed oil with carnosic acid during thermal degradation at 60 °C (**A_1_**,**B_1_**,**C_1_**,**D_1_**,**E_1_**) and 100 °C (**A_2_**,**B_2_**,**C_2_**,**D_2_**,**E_2_**).

The apparent viscosities of RSO were increased after 0, 8, 16, 24, and 32 h of degradation, as shown in Figure 1. With the increasing addition of CA, the viscosities of RSO were decreased. Also, it showed that the viscosity was increased with the increasing shear rate, indicating that all the RSO samples with no additives present Newtonian behavior during thermal degradation.

**Figure 2:**
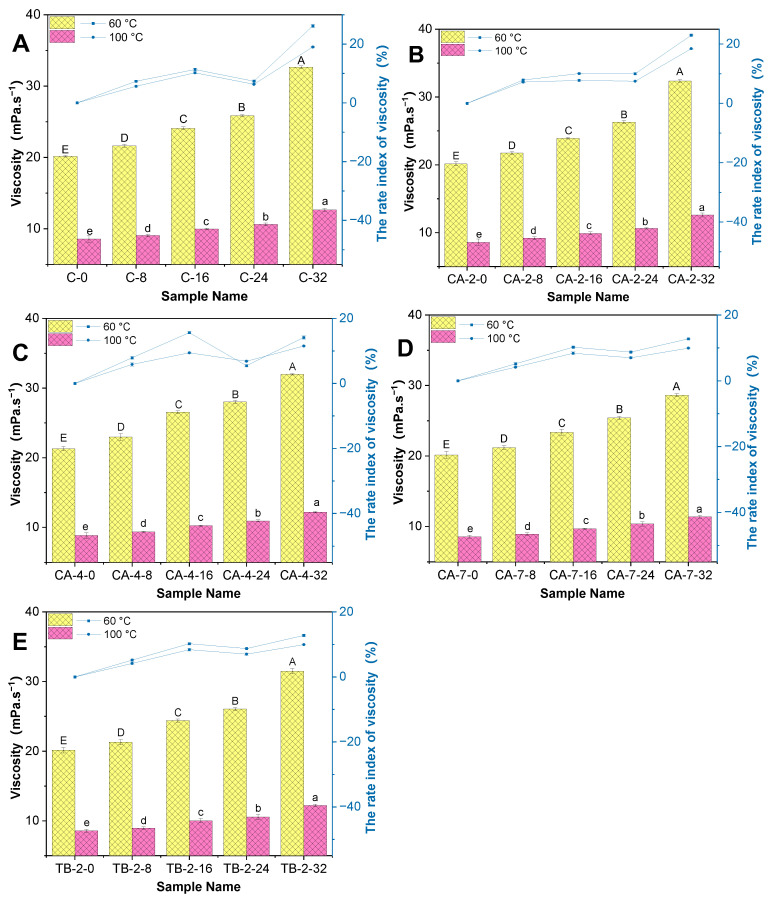
The apparent viscosity and the rate index of viscosity conducted for samples during thermal degradation (60 °C and 100 °C), (**A**–**E**). Different letters in the same column superscripted on the results are significantly different (*p* < 0.05).

The viscosities and the rate index of RSO are shown in Figure 2. The viscosity of fresh RSO (control, C) increased intensively with the extension of thermal degradation. The viscosity was 20.15 ± 0.10, 21.63 ± 0.22, 24.09 ± 0.25, 25.87 ± 0.17, and 32.64 ± 0.23 mPa·s^−1^ (*p* < 0.05) at the time of 0, 8, 16, 24, and 32 h at 60 °C, respectively. The increasing oil viscosity with the extensive thermal degradation in all samples was consistent with palm oil and soybean oil [32]. The extension of thermal degradation can increase the chain length and increase saturated triglyceride fatty acid, which forms a higher-molecular-weight compound and can have a significant influence on the viscosity of vegetable oil [33]. The viscosity of the frying oil increased due to the polymerization reactions and played a dominant role in the oil adherence to the fried products [34]. The degradation of oil forms some surface-active polar compounds and can reduce the interfacial tension and lead to oil uptake [35]. The results indicated that the control RSO exhibited the highest level of viscosity among the samples with CA and TBHQ tested. Upon the incorporation of CA and TBHQ in the RSO, there was a decrease in the rate of viscosity growth observed. Notably, the sample with a concentration of 700 mg/kg of CA demonstrated the lowest viscosity of all the samples analyzed after 32 h of degradation. These findings suggest that the proper ratio and concentration of CA can enhance and extend the utilization of RSO with low viscosities [36].

### 3.2. The Dependency of Viscosity on Temperature

**Figure 3:**
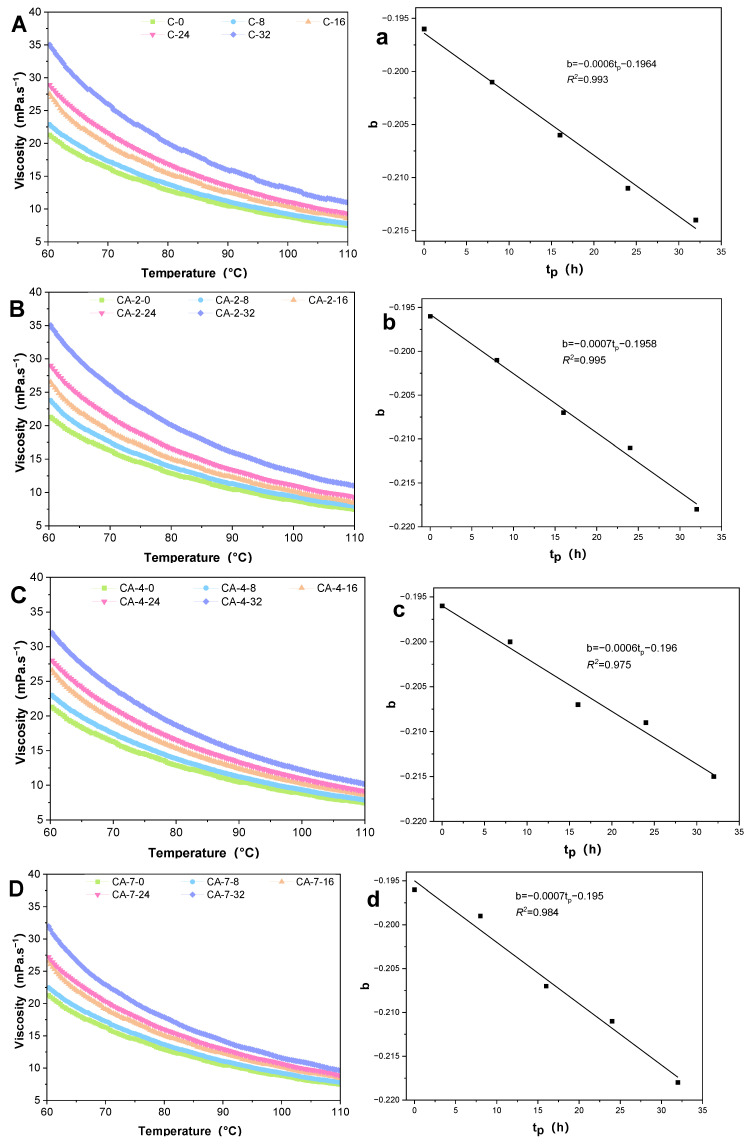

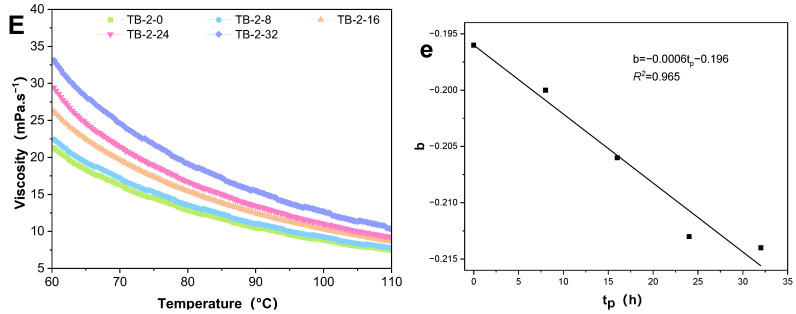
The viscosity versus temperature of samples after thermal degradation (**A**–**E**). The regression analysis between parameters of *Lioumbas* model *b* value during thermal degradation (*t_P_*, h; **a**–**e**).

The correlation between viscosity and temperature in oils was observed, particularly during thermal degradation. Figure 3A–E demonstrate that the viscosity of RSO with CA decreased non-linearly with the increasing temperature in an exponential condition. The highest viscosity of RSO was recorded for the samples that underwent 32 h of degradation, whereas the lowest viscosity was noted in the control samples processed for 0 h. This phenomenon can be attributed to the acceleration of molecular motion and reduction of intermolecular forces in large molecular materials, such as those present in food, as a result of elevated temperatures and their contribution to rheological behavior [37]. The viscosity of RSO can reflect the degradation of the inner molecular composition [38]. The addition of CA and TBHQ can delay the degradation of RSO in viscosity during thermal degradation to a certain extent.

To perform a deeper analysis, an investigation was conducted to determine if there exists an equation that can characterize the dependence of viscosity on temperature. The equations of the *Lioumbas* model (Equation (1)) and *Arrhenius* model (Equation (2)) were used to calculate. The accuracy of Equation (1) was *R*^2^ ≥ 0.998, while the accuracy of Equation (2) was *R*^2^ ≥ 0.812, as can be seen in Table 1. Both of these models provided a better fit for the viscosity data. In contrast, the *Lioumbas* model demonstrated an excellent fit for the dependence of viscosity with *R*^2^ ≥ 0.998. Therefore, the *Lioumbas* model was deemed to be more appropriate for analyzing the dependence of viscosity on temperature and was used for the subsequent analysis.

In Table 1, the parameters show that the *a* value was related to the viscosity of RSO when *T_m_* = 1 °C, and the *b* value was negative, indicating that the viscosity changed with the temperature. After 32 h of thermal degradation, the *a* value changed from 6.32 ± 0.01 to 7.10 ± 0.01 with the fresh RSO, while the *a* value had changed from 6.23 ± 0.01 to 7.19 ± 0.02 in the RSO with 700 mg/kg CA. All the samples showed that the *a* value had significantly increased with the thermal degradation.

Additionally, all the samples showed a decrease in the *b* value during thermal degradation. The absolute ‘*b*’ value (original value is negative) was similar to ‘*Ea*’ in the *Arrhenius* model, indicating the change rate of viscosity with temperature and that the reactive molecule reaches the minimum energy required to activate the molecule. After multiple quasi-linear regressions in the *Lioumbas* model, the *b* value decreased linearly with thermal processing (*t_P_*, h) in all samples (*R*^2^ ≥ 0.965), as shown in Figure 3a–e. It indicated that the viscosity regression coefficient *b* value correlated with thermal degradation and the viscosity parameters.

**Figure 4:**
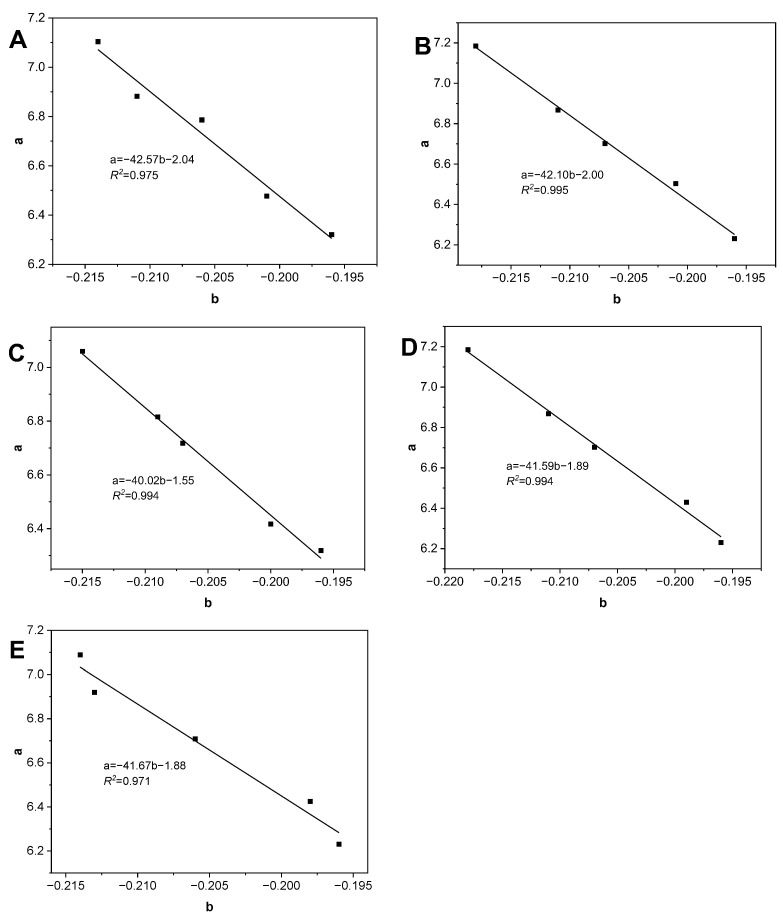
The regression analysis between the parameters of *Lioumbas* model *b* value versus *a* value during thermal degradation (**A**: C; **B**: CA-2; **C**: CA-4; **D**: CA-7; **E**: TB-2).

The regression analysis between the parameters in the *Lioumbas* model *b* value and *a* value during thermal degradation is shown in Figure 4. It can be seen that the *a* value decreased linearly (*R*^2^ ≥ 0.971) with the increase in the *b* value, and there was a negative correlation between the *a* value and *b* value. Compared with the control samples, the slope coefficient was increased with CA and TBHQ concentrations (from −42.57 to −41.67). It showed that there is a potential association between the concentrations of CA and the viscosity parameters with thermal degradation.

### 3.3. The Chemical Composition of Rapeseed Oil

The chemical composition plays a critical role in the thermal oxidation of oils. As a result of continuous heating, there is a dramatic alteration in the FA composition of the oil. This study investigated the effect of different concentrations of CA and TBHQ on the FA composition by measuring it at intervals during thermal processing. The results of this study provide insight into the relationship between the chemical composition and thermal oxidation of oils.

As shown in Table 2, the predominant component of RSO was oleic acid (62.43 ± 0.33 mg/100 mg). During heating, the FA compositions of all fresh RSO samples showed a decrease in unsaturated fatty acids (UFAs), especially in polyunsaturated fatty acids (PUFAs). The oxidation process was observed to increase saturation, and the increase was attributed to the formation of palmitic, stearic, and oleic acid from linoleic and linolenic acid in RSO with all samples. The linolenic acid in the control samples was 7.46 ± 0.15 mg/100 mg, while it was 4.91 ± 0.10 mg/100 mg after 32 h of thermal degradation. The attrition rate was nearly 34.18%. Interestingly, the decrease rate of PUFAs was 18.08%, 18.94%, 16.50%, 13.84%, and 18.15% in the C, CA-2, CA-4, CA-7, and TB-2 samples after 32 h of degradation. This showed that the addition of CA and TBHQ could modify the RSO properties, especially the FA compositions. The addition of 700 mg/kg carnosic acid (CA-7 samples) showed the most effective means to prevent fatty acid degradation during heating. CA could perform the best in inhibiting thermal oxidation during thermal degradation [39].

The results of the ECN values for RSO, as presented in Table 3, showed a similar trend to the FA composition. The ECN value of RSO was observed to increase significantly with heating, increasing the saturation levels, specifically for palmitic and stearic acid, and it decreased in the levels of PUFAs, such as linoleic and linolenic acid. This indicates that the oxidation of RSO involves a free-radical chain reaction and accelerates the oxidation of unsaturated fatty acids [40]. Also, the ECN value can be used as an accurate method to predict the viscosities of TAG from the chemical composition [41]. Furthermore, the presence of CA as a source of natural antioxidants could eliminate consumer concerns compared with synthetic antioxidants [42].

### 3.4. The Total Polar Compounds (TPC) of Rapeseed Oil

The results of the TPC value showed a significant increase after 32 h of thermal degradation at 180 °C (Table 3). The formation of polar compounds is a result of hydrolysis, oxidation, and polymerization reactions that occur during thermal degradation [5]. With the antioxidant concentrations increasing, the TPC values increased slowly, especially with the addition of CA. After 180 °C and 32 h of thermal degradation, the addition of 200 mg/kg of TBHQ (TB-2) and 200 mg/kg of carnosic acid (CA-2) to RSO showed no significant difference in TPC values, which were 32.50 ± 0.50% and 32.00 ± 0.50%, respectively. This indicated that the concentration of CA and TBHQ played a crucial role in thermal oxidation. After 32 h of thermal degradation, the lowest TPC value was found in the CA-7 sample, at 26.00 ± 0.50%, which was under 27%. It meant that the addition of CA played a crucial role in thermal oxidation, extending the usage life of oil and reducing the efficiency of antioxidants [43]. The addition of 700 mg/kg CA can effectively delay the rapid growth of TPC value and extend the waste point of RSO.

### 3.5. The Model Regression of Rapeseed Oil

The multiple linear regression analysis was adopted to obtain the parameters in Equation (1) to forecast the thermal degradation of RSO. The parameters (*a* and *b*) in the *Lioumbas* model were measured after 32 h of thermal degradation (samples taken every 8 h). The results showed that the parameters fit well in the regressions and the experimental data. In particular, the *a* value depends linearly on the *b* value in Figure 4:

C: *a* = −42.57*b* − 2.04, *R*^2^ = 0.975 (4)

*b* = −0.0006*t_P_*− 0.1964, *R*^2^ = 0.993 (5)

CA-2: *a* = −42.10*b* − 2.00, *R*^2^ = 0.995 (6)

*b* = −0.0007*t_P_*− 0.1958, *R*^2^ = 0.995 (7)

CA-4: *a* = −42.02*b* − 1.55, *R*^2^ = 0.994 (8)

*b* = −0.0006*t_P_*− 0.1960, *R*^2^ = 0.975 (9)

CA-7: *a* = −41.59*b* − 1.89, *R*^2^ = 0.994 (10)

*b* = −0.0007*t_P_*− 0.1950, *R*^2^ = 0.984 (11)

TB-2: *a* = −41.67*b* − 1.88, *R*^2^ = 0.971 (12)

*b* = −0.0006*t_P_*−0.1960, *R*^2^ = 0.965 (13)

**Figure 5:**
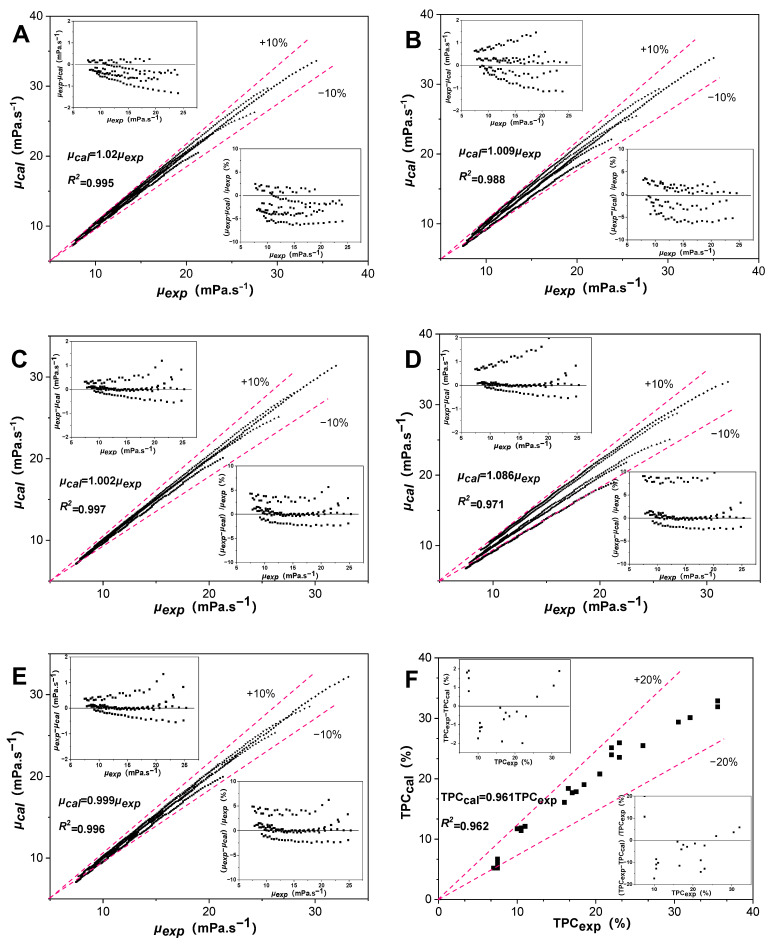
The relation of viscosity between the calculated values and those experimental values from the *Lioumbas* model during thermal degradation (**A**: C; **B**: CA-2; **C**: CA-4; **D**: CA-7; **E**: TB-2). The relation of TPC between the calculated values and those experimental values during thermal degradation (**F**). The insert figures show that the actual difference in viscosity/TPC values (top) and the deviation% (bottom) between calculated values and the experimental values.

The parity plot between the calculated viscosity (*μ_cal_*) and the experimental data (*μ_exp_*) by the *Lioumbas* model during thermal degradation was analyzed using Equation (1), together with Equations (4)–(13), in Figure 5A–E. The top figure shows the experimental data minus the calculated data versus the experimental data, and the bottom shows the deviation% between the calculated data and the model calculated predictions. It measures the *μ_exp_-μ_cal_* of all tested samples to be less than ±2 mPa·s^−1^ from the top figures in the plots. In particular, the maximum deviation% is about 10% in Figure 5D, with the addition of 700 mg/kg CA (CA-7). This deviation highlights the impact of the CA concentration on the viscosity fluctuation of RSO during thermal degradation. Overall, the calculated predictions of viscosity showed an accuracy of less than ±10% for all the test samples.

Multiple linear regression was used to analyze the correlation between the parameters of Equation (1) and the chemical composition of RSO during thermal degradation. Both the TPC value and the ECN were used to measure the chemical composition of RSO. The TPC value is directly related to the quality of RSO and the ECN is the molecular conformation of TAG in FA composition, which was analyzed by Equation (3). The results showed that the correlation between the *a* value and *b* value is the same trend in Equations (4), (6), (8), (10) and (12), and the equation is as follows:

*b* = 0.2 − 0.024ECN − 0.001TPC, *R*^2^ = 0.947 (14)

The linear regression analysis for the thermal degradation and the TPC value of RSO with CA and TBHQ are shown as follows:

C: TPC = 0.8625*t_P_* + 5.2, *R*^2^ = 0.959 (15)

CA-2: TPC = 0.8438*t_P_* + 4.9, *R*^2^ = 0.930 (16)

CA-4: TPC = 0.7313*t_P_* + 6.0, *R*^2^ = 0.983 (17)

CA-7: TPC = 0.5875*t_P_* + 6.7, *R*^2^ = 0.992 (18)

TB-2: TPC = 0.7625*t_P_* + 5.6, *R*^2^ = 0.963 (19)

The parity plot between calculated TPC_cal_ values and the experimental TPC_exp_ values in all samples during thermal degradation was analyzed using Equations (15)–(19) in Figure 5F. The top figure shows the experimental data minus the calculated data versus the experimental data, and the bottom shows the deviation% between the calculated data and the calculated predictions. The TPC_exp_-TPC_cal_ values of all tested samples were less than ±2 % from the top figures in the plots. The deviation% in the equations used to predict the TPC value was less than ±20% in all test samples in the bottom. There may be some limitations to the model, depending on the type of cooking oil and the amounts of antioxidants in the oil.

### 3.6. Pearson Correlation of Rapeseed Oil

**Figure 6:**
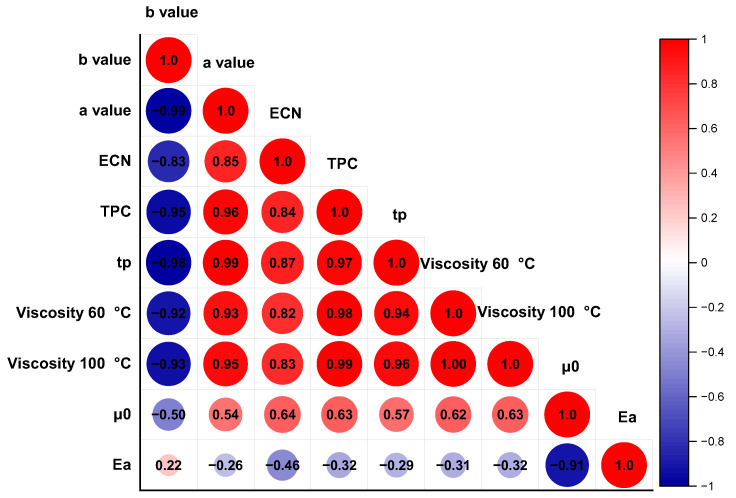
The *Pearson* correlation analysis of the chemical composition, total polar compounds, and viscosity in rapeseed oil with carnosic acid during thermal degradation.

The *Pearson* correlation analysis of the chemical composition, total polar compounds, and viscosity in RSO with antioxidants during thermal degradation is shown in Figure 6. The red color shows a positive relationship, and the blue one shows a negative relationship. And the area in the figure is related to the coefficient of association. Compared with the parameters in the *Arrhenius* model (*μ*_0_ and *E_a_*), the parameters in the *Lioumbas* model (*a* and *b* value) showed a better correlation with the chemical composition, TPC, and the viscosity of RSO during thermal degradation. Based on the *Pearson* correlation, the *a* value, ECN, TPC, *t_P_*, viscosity, and *μ*_0_ showed a positive relationship in RSO during thermal degradation. In particular, the higher the TPC, the longer the *t_P_* (*r* = 0.97, *p* < 0.01), the higher the ECN (*r* = 0.84, *p* < 0.05), and the higher viscosity at 60 °C and 100 °C (*r* = 0.98, *r* = 0.99, *p* < 0.01). The ECN value showed that the UFAs decreased and the saturation of oil increased with the heating in Table 2 and Table 3. Meanwhile, there was a negative correlation between the *b* value and *a* value (*p* < 0.01), and this was consistent with the results shown in Figure 4. In conclusion, the increased TPC value, ECN, and viscosity; the extended period of thermal processing, and the degradation of UFAs of RSO with free radical chain reaction showed a prominent of oxidative reaction [44].

## 4. Conclusions

The temperature-dependent behavior of rapeseed oil (RSO) with carnosic acid showed a better fit in the *Lioumbas* model (*R*^2^ ≥ 0.999). The parameter *b* in the *Lioumbas* model showed a decrease linearly with thermal processing (*t_P_*, *R*^2^ ≥ 0.965). The addition of 700 mg/kg carnosic acid (CA) showed the lowest degradation of polyunsaturated fatty acids, i.e., 13.84%. The multiple nonlinear regression analysis predicted that the viscosity was less than ±2 mPa·s^−1^ and the deviation% was less than ±10%, and the predicted total polar compound was less than ±2% and the deviation% was less than ±20% in all the samples. The total polar compound showed a positive relationship with the thermal processing time (*t_P_*, *r* = 0.97, *p* < 0.01), effective carbon numbers (*r* = 0.84, *p* < 0.05), and viscosity (*r* = 0.98, *r* = 0.99, *p* < 0.01). The results suggest that the total polar compound, chemical composition, effective carbon numbers, and viscosity parameters can be potentially used as a basis for the development of models to identify the degradation and make a potential prediction for the stability of rapeseed oil. The above model can be applied to frying oil, especially for fast-food enterprises in relation to frying oil quality control.

## Figures and Tables

**Table 1:** Effects of the parameters of the *Lioumbas* and *Arrhenius* models for rapeseed oil with carnosic acid on viscosity in different states of thermal degradation *^A,B^*.

		C			CA-2			CA-4			CA-7			TB-2		
*Lioumbas* model	Conditions/h	*a*	*b*	*R* ^2^	*a*	*b*	*R* ^2^	*A*	*b*	*R* ^2^	*a*	*b*	*R* ^2^	*a*	*b*	*R* ^2^
	0	6.32 ± 0.01 ^e^	−0.19 ± 0.01 ^d^	0.999	6.23 ± 0.01 ^e^	−0.20 ± 0.01 ^c^	0.999	6.32 ± 0.02 ^e^	−0.19 ± 0.01 ^b^	0.999	6.23 ± 0.01 ^e^	−0.19 ± 0.01 ^d^	0.999	6.23 ± 0.01 ^e^	−0.20 ± 0.01 ^b^	0.999
	8	6.48 ± 0.01 ^d^	−0.20 ± 0.01 ^c^	1.000	6.50 ± 0.01 ^d^	−0.20 ± 0.01 ^c^	0.999	6.42 ± 0.02 ^d^	−0.20 ± 0.02 ^b^	1.000	6.43 ± 0.02 ^d^	−0.20 ± 0.01 ^c^	1.000	6.43 ± 0.01 ^d^	−0.20 ± 0.01 ^b^	1.000
	16	6.79 ± 0.02 ^c^	−0.21 ± 0.01 ^b^	0.998	6.70 ± 0.02 ^c^	−0.21 ± 0.01 ^b^	0.999	6.72 ± 0.01 ^c^	−0.21 ± 0.01 ^b^	0.999	6.70 ± 0.02 ^c^	−0.21 ± 0.02 ^b^	0.999	6.71 ± 0.01 ^c^	−0.21 ± 0.02 ^a^	1.000
	24	6.88 ± 0.01 ^b^	−0.21 ± 0.01 ^b^	1.000	6.87 ± 0.01 ^b^	−0.21 ± 0.02 ^b^	0.999	6.82 ± 0.01 ^b^	−0.21 ± 0.01 ^b^	1.000	6.87 ± 0.02 ^b^	−0.21 ± 0.01 ^b^	1.000	6.92 ± 0.01 ^b^	−0.21 ± 0.01 ^a^	0.999
	32	7.10 ± 0.01 ^a^	−0.22 ± 0.01 ^a^	1.000	7.19 ± 0.01 ^a^	−0.22 ± 0.02 ^a^	1.000	7.06 ± 0.01 ^a^	−0.22 ± 0.01 ^a^	1.000	7.19 ± 0.02 ^a^	−0.22 ± 0.02 ^a^	0.999	7.09 ± 0.02 ^a^	−0.22 ± 0.01 ^a^	1.000
*Arrhenius* model	Conditions/h	*μ*_0_	*E_a_*	*R* ^2^	*μ*_0_	*E_a_*	*R* ^2^	*μ*_0_	*E_a_*	*R* ^2^	*μ*_0_	*E_a_*	*R* ^2^	*μ*_0_	*E_a_*	*R* ^2^
	0	0.04 ± 0.01 ^c^	16,684.93 ± 2.22 ^c^	0.935	0.04 ± 0.01 ^b^	16,684.93 ± 2.96 ^c^	0.935	0.04 ± 0.01 ^b^	16,684.93 ± 7.21 ^d^	0.935	0.04 ± 0.01 ^c^	16,684.93 ± 3.97 ^c^	0.935	0.04 ± 0.01 ^d^	16,684.93 ± 7.33 ^b^	0.935
	8	0.04 ± 0.01 ^c^	17,161.38 ± 3.76 ^b^	0.935	0.05 ± 0.02 ^a^	16,595.06 ± 6.22 ^c^	0.925	0.03 ± 0.01 ^c^	17,916.81 ± 5.66 ^b^	0.953	0.06 ± 0.01 ^b^	15,756.17 ± 4.73 ^d^	0.912	0.05 ± 0.01 ^c^	16,425.37 ± 11.36 ^c^	0.925
	16	0.04 ± 0.01 ^c^	17,679.87 ± 3.55 ^a^	0.933	0.04 ± 0.02 ^b^	17,343.72 ± 3.43 ^b^	0.930	0.04 ± 0.01 ^b^	17,556.30 ± 6.86 ^c^	0.936	0.04 ± 0.01 ^bc^	17,343.72 ± 6.61 ^b^	0.930	0.03 ± 0.02 ^d^	18,052.69 ± 10.51 ^a^	0.949
	24	0.11 ± 0.01 ^b^	14,825.43 ± 4.76 ^d^	0.858	0.05 ± 0.03 ^a^	16,752.26 ± 5.51 ^c^	0.910	0.03 ± 0.01 ^c^	18,168.82 ± 10.21 ^a^	0.948	0.04 ± 0.01 ^bc^	17,587.39 ± 7.72 ^a^	0.936	0.07 ± 0.01 ^b^	15,994.97 ± 6.90 ^d^	0.886
	32	0.18 ± 0.01 ^a^	13,704.19 ± 4.11 ^e^	0.812	0.03 ± 0.03 ^c^	18,683.22 ± 4.44 ^a^	0.939	0.12 ± 0.01 ^a^	14,762.37 ± 5.92 ^e^	0.839	0.09 ± 0.02 ^a^	15,588.09 ± 5.59 ^d^	0.860	0.12 ± 0.01 ^a^	14,842.00 ± 5.11 ^e^	0.845

*^A^* Different letters in the same column superscripted on the results are significantly different (*p* < 0.05). *^B^* Values present mean ± standard derivatives of three triplicates

**Table 2:** The fatty acid (FA) compositions (%) of rapeseed oil with carnosic acid and TBHQ during thermal degradation.

FA	C16:0 Palmitic Acid	C16:1 Palmitoleic Acid	C18:0 Stearic Acid	C18:1 Oleic Acid	C18:2 Linoleic Acid	C20:0 Arachidic Acid	C18:3n3 α-Linolenic Acid	C22:0 Behenic Acid	UFAs	MUFAs	PUFAs
C-0	4.51 ± 0.02	0.15 ± 0.01	1.49 ± 0.02	62.43 ± 0.33	20.36 ± 0.27	0.45 ± 0.01	7.46 ± 0.15	0.24 ± 0.01	90.40 ± 0.76	62.58 ± 0.34	27.82 ± 0.42
C-16	4.83 ± 0.10	0.17 ± 0.01	1.62 ± 0.02	64.30 ± 0.22	19.03 ± 0.14	0.49 ± 0.01	6.28 ± 0.36	0.30 ± 0.01	89.78 ± 0.73	64.47 ± 0.23	25.31 ± 0.50
C-32	5.13 ± 0.34	0.21 ± 0.01	1.80 ± 0.05	66.11 ± 0.38	17.88 ± 0.02	0.53 ± 0.01	4.91 ± 0.10	0.32 ± 0.01	89.11 ± 0.51	66.32 ± 0.39	22.79 ± 0.12
CA-2-0	4.51 ± 0.02	0.15 ± 0.01	1.49 ± 0.02	62.43 ± 0.33	20.36 ± 0.27	0.45 ± 0.01	7.46 ± 0.15	0.24 ± 0.01	90.40 ± 0.76	62.58 ± 0.34	27.82 ± 0.42
CA-2-16	4.79 ± 0.02	0.16 ± 0.01	1.61 ± 0.02	63.49 ± 0.20	19.62 ± 0.10	0.49 ± 0.01	6.54 ± 0.30	0.28 ± 0.01	89.81 ± 0.61	63.65 ± 0.21	26.16 ± 0.40
CA-2-32	5.37 ± 0.36	0.18 ± 0.01	1.77 ± 0.10	66.31 ± 0.58	17.77 ± 0.24	0.56 ± 0.01	4.78 ± 0.23	0.32 ± 0.01	89.04 ± 1.06	66.49 ± 0.59	22.55 ± 0.47
CA-4-0	4.51 ± 0.02	0.15 ± 0.01	1.49 ± 0.02	62.43 ± 0.33	20.36 ± 0.27	0.45 ± 0.01	7.46 ± 0.15	0.24 ± 0.01	90.40 ± 0.76	62.58 ± 0.34	27.82 ± 0.42
CA-4-16	4.76 ± 0.05	0.17 ± 0.01	1.61 ± 0.01	63.47 ± 0.54	19.52 ± 0.19	0.48 ± 0.01	6.78 ± 0.32	0.28 ± 0.01	89.94 ± 1.06	63.64 ± 0.55	26.30 ± 0.51
CA-4-32	5.02 ± 0.25	0.18 ± 0.01	1.70 ± 0.01	66.42 ± 0.66	17.92 ± 0.34	0.51 ± 0.01	5.31 ± 0.22	0.30 ± 0.01	89.83 ± 1.23	66.60 ± 0.67	23.23 ± 0.56
CA-7-0	4.51 ± 0.02	0.15 ± 0.01	1.49 ± 0.02	62.43 ± 0.33	20.36 ± 0.27	0.45 ± 0.01	7.46 ± 0.15	0.24 ± 0.01	90.40 ± 0.76	62.58 ± 0.34	27.82 ± 0.42
CA-7-16	4.58 ± 0.06	0.16 ± 0.01	1.68 ± 0.01	63.96 ± 0.34	19.60 ± 0.05	0.46 ± 0.01	6.60 ± 0.21	0.24 ± 0.01	90.32 ± 0.61	64.12 ± 0.35	26.20 ± 0.26
CA-7-32	5.01 ± 0.05	0.17 ± 0.01	1.78 ± 0.01	65.41 ± 0.52	18.23 ± 0.16	0.50 ± 0.01	5.74 ± 0.10	0.33 ± 0.01	89.55 ± 0.79	65.58 ± 0.53	23.97 ± 0.26
TB-2-0	4.51 ± 0.02	0.15 ± 0.01	1.49 ± 0.02	62.43 ± 0.33	20.36 ± 0.27	0.45 ±0.01	7.46 ± 0.15	0.24 ± 0.01	90.40 ± 0.76	62.58 ± 0.34	27.82 ± 0.42
TB-2-16	4.87 ± 0.06	0.15 ± 0.01	1.60 ± 0.01	64.16 ± 0.58	19.15 ± 0.43	0.50 ±0.01	6.41 ± 0.35	0.26 ± 0.01	89.87 ± 1.37	64.31 ± 0.59	25.56 ± 0.78
TB-2-32	5.16 ± 0.02	0.17 ± 0.01	1.72 ± 0.01	66.36 ± 0.44	17.64 ± 0.58	0.55 ± 0.01	5.13 ± 0.26	0.33 ± 0.01	89.30 ± 1.29	66.53 ± 0.45	22.77 ± 0.84

C-0, the control RSO thermal for 0 h; C-16, the control RSO thermal for 16 h; C-32, the control RSO thermal for 32 h; CA-2-0, 200 mg/kg CA in RSO (CA-2) heating for 0 h was simplified as CA-2-0; CA-2-16, 200 mg/kg CA in RSO (CA-2) heating for 16 h; CA-2-32, 200 mg/kg CA in RSO (CA-2) heating for 32 h; CA-4-0, 400 mg/kg CA in RSO (CA-4) heating for 0 h; CA-4-16, 400 mg/kg CA in RSO (CA-4) heating for 16 h; CA-4-32, 400 mg/kg CA in RSO (CA-4) heating for 32 h; CA-7-0, 700 mg/kg CA in RSO (CA-7) heating for 0 h; CA-7-16, 700 mg/kg CA in RSO (CA-7) heating for 16 h; CA-7-32, 700 mg/kg CA in RSO (CA-7) heating for 32 h; TB-2-0, 200 mg/kg TBHQ in RSO (TB-2) heating for 0 h; TB-2-16, 200 mg/kg TBHQ in RSO (TB-2) heating for 16 h; TB-2-32, 200 mg/kg TBHQ in RSO (TB-2) heating for 32 h.

**Table 3:** The calculated effective carbon numbers (ECNs) and the total polar compounds (TPC) of rapeseed oil with carnosic acid during thermal degradation *^A^*^,*B*^.

ECN (Dimensionless)					
Conditions/h	C	CA-2	CA-4	CA-7	TB-2
0	16.13 ± 0.01 ^c^	16.14 ± 0.01 ^c^	16.13 ± 0.01 ^e^	16.13 ± 0.01 ^b^	16.13 ± 0.01 ^a^
8	16.17 ± 0.01 ^b^	16.18 ± 0.01 ^b^	16.13 ± 0.01 ^d^	16.20 ± 0.01 ^a^	16.18 ± 0.01 ^b^
16	16.17 ± 0.02 ^b^	16.15 ± 0.01 ^c^	16.17 ± 0.01 ^c^	16.20 ± 0.01 ^a^	16.18 ± 0.01 ^b^
24	16.21 ± 0.01 ^a^	16.18 ± 0.01 ^b^	16.21 ± 0.01 ^b^	16.20 ± 0.01 ^a^	16.21 ± 0.01 ^a^
32	16.23 ± 0.01 ^a^	16.22 ± 0.01 ^a^	16.26 ± 0.01 ^a^	16.22 ± 0.01 ^a^	16.22 ± 0.01 ^a^
**TPC (%)**					
0	7.00 ± 0.50 ^e^	7.50 ± 0.50 ^e^	7.50 ± 0.50 ^e^	7.50 ± 0.50 ^e^	7.50 ± 0.50 ^e^
8	11.00 ± 0.50 ^d^	10.50 ± 0.50 ^d^	10.50 ± 0.50 ^d^	10.50 ± 0.50 ^d^	10.00 ± 0.50 ^d^
16	18.50 ± 0.50 ^c^	16.50 ± 0.50 ^c^	17.00 ± 0.50 ^c^	16.00 ± 0.50 ^c^	17.50 ± 0.50 ^c^
24	23.00 ± 0.50 ^b^	22.00 ± 0.50 ^b^	23.00 ± 0.50 ^b^	20.50 ± 0.50 ^b^	22.00 ± 0.50 ^b^
32	35.50 ± 0.50 ^a^	32.50 ± 0.50 ^a^	30.50 ± 0.50 ^a^	26.00 ± 0.50 ^a^	32.00 ± 0.50 ^a^

*^A^* Different letters in the same column superscripted on the results are significantly different (*p* < 0.05). *^B^* Values present mean ± standard derivatives of three triplicates.

## Data Availability

The data presented in this study are available on request from the corresponding author.

## Abbreviations

RSO	rapeseed oil
ECN	effective carbon numbers
TPC	total polar compounds
*t_P_*	processing time (h)
C	control samples
CA	carnosic acid
TBHQ	*tert*-Butylhydroquinone
FA	fatty acid
*b*	constant data in the *Lioumbas* model
*a*	constant data in the *Lioumbas* model
*μ*_0_	constant data in the *Arrhenius* model
*E_a_*	activation energy in the *Arrhenius* model
*R*	universal gas constant, in the *Arrhenius* model
*T_m_*	measured temperature
TAG	triacylglycerol
UFAs	unsaturated fatty acids
MUFAs	monounsaturated fatty acids
PUFAs	polyunsaturated fatty acids
